# Neutrophil recruitment and leukocyte response following focused ultrasound and microbubble mediated blood-brain barrier treatments

**DOI:** 10.7150/thno.52710

**Published:** 2021-01-01

**Authors:** Charissa Poon, Carly Pellow, Kullervo Hynynen

**Affiliations:** 1 Physical Sciences Platform, Sunnybrook Research Institute, Toronto, Ontario, Canada.; 2 Institute of Biomedical Engineering, University of Toronto, Toronto, Ontario, Canada.; 3 Department of Medical Biophysics, University of Toronto, Toronto, Ontario, Canada.

**Keywords:** focused ultrasound, microbubbles, neutrophils, blood-brain barrier, two-photon fluorescence microscopy

## Abstract

**Rationale:** Delivery of therapeutic agents to the brain is limited by the presence of the blood-brain barrier (BBB). An emerging strategy to temporarily and locally increase the permeability of the BBB is the use of transcranial focused ultrasound (FUS) and systematically injected microbubbles (MBs). FUS+MB BBB treatments cause an acute inflammatory response, marked by a transient upregulation of pro-inflammatory genes; however, the cellular immune response remains unknown.

**Methods:** FUS+MB BBB treatments were monitored in real-time using two-photon fluorescence microscopy and transgenic EGFP Wistar rats, which harbour several fluorescent cell types. Leukocyte identification and counts were confirmed using magnetic resonance imaging-guided FUS+MB BBB treatments. Participation of leukocytes in reducing β-amyloid pathology following repeated FUS+MB BBB treatments was investigated in the TgCRND8 mouse model of Alzheimer's disease.

**Results:** Intravascular leukocyte activity indicative of acute inflammation were identified, including transendothelial migration, formation of cell aggregates, and cell masses capable of perturbing blood flow. Leukocyte responses were only observed after the onset of sonication. Neutrophils were identified to be a key participating leukocyte. Significantly more neutrophils were detected in the sonicated hemisphere compared to the contralateral hemisphere, and to untreated controls. Three to five biweekly FUS+MB BBB treatments did not induce significantly more neutrophil recruitment, nor neutrophil phagocytosis of β-amyloid plaques, in TgCRND8 mice compared to untreated controls.

**Conclusions:** This study provides evidence that the cellular aspect of the peripheral immune response triggered by FUS+MB BBB treatments begins immediately after sonication, and emphasizes the importance for further investigations to be conducted to understand leukocyte dynamics and cerebral blood flow responses to FUS+MB BBB treatments.

## Introduction

Drug delivery to the brain is limited by the blood-brain barrier (BBB), which prevents the entry of over 98% of small-molecule drugs 1. Substances that can cross the BBB via transmembrane diffusion generally have molecular masses under 400-500 Da and high lipid solubility 1. Vascular endothelial cells (ECs) can also transport substances such as glucose and amino acids through transporter complexes located on the luminal or abluminal surfaces of the ECs 2, but have notably reduced endocytosis in the BBB compared to peripheral endothelia 1.

An emerging strategy to temporarily and locally increase BBB permeability for targeted drug delivery is to use transcranial focused ultrasound (FUS) in conjunction with preformed, systemically circulating microbubbles (MBs). In this approach, during FUS exposure, MBs oscillate in the vasculature and exert mechanical stresses on blood vessel endothelia, resulting in increased paracellular and transcellular transport. In addition to enhanced agent delivery 3-8, FUS+MB exposure has been shown to induce biological effects such as neurogenesis in the dentate gyrus 9, 10, glial cell activation 11, and a reduction in β-amyloid (Aβ) plaque load in transgenic mouse models of Alzheimer's disease (AD) 12-15.

The mechanisms by which FUS+MB BBB treatments clear Aβ pathology have yet to be fully elucidated. While FUS+MB BBB treatments have been shown to induce glial cell activation and phagocytosis of Aβ plaques 11, 13, there is likely a saturation limit 11. In addition, microglia, the resident macrophages in the CNS, are poor phagocytes compared to peripheral immune cells, in part due to the lack of certain cell surface antigens that facilitate immune responses, such as major histocompatibility class II molecules and CD45 16. Infiltration of peripheral blood monocytes following FUS+MB BBB treatments has been previously reported; however, such events were only observed coincident with vascular damage, such as clusters of erythrocyte extravasation 17 or intracerebral hemorrhage 18. It is possible that peripheral immune cells have not been previously detected in FUS+MB BBB treatments that did not result in damage due to the small number of participating cells, and the limits of detection of the assays used. FUS+MB treatments delivered using a clinical imaging dose of MBs have been shown to result in an acute inflammatory response, characterized by an upregulation of pro-inflammatory genes, such as *Sele*, *Cxcl1*, and *Ccl3*, at 6 h following treatment 19, 20. In addition, use of FUS+MB to increase blood-tumor barrier permeability has been observed to induce a mild and transient response in dendritic cells 21. It is hypothesized that a cellular aspect of acute inflammatory responses, specifically the recruitment of peripheral immune cells, would be induced by FUS+MB BBB treatments as well.

In this paper, the recruitment of peripheral immune cells induced by FUS+MB BBB treatments and their role in FUS+MB mediated Aβ plaque clearance was investigated. To observe the response of vasculature and cells to FUS+MB treatments in real-time, transgenic EGFP Wistar rats which harbour several fluorescent cell types were monitored *in vivo* using two-photon fluorescence microscopy (2PFM). This study reveals a previously unreported cellular component of the acute inflammatory response that results from FUS+MB mediated BBB treatments, which may be useful in informing future studies involving neuroinflammatory disease conditions (e.g. AD) or in consideration of using FUS+MB BBB treatments for drug delivery. This study is relevant as clinical trials involving the use of FUS+MB BBB treatments are currently underway (ClinicalTrials.gov identifier: NCT04118764).

## Materials and methods

There were three arms to this study: (A) *In vivo* 2PFM monitoring of peripheral immune cell response to FUS+MB BBB treatments, (B) Identification and quantification of participating peripheral immune cells using immunohistochemistry, and (C) Investigation into the potential of the identified immune cells in assisting in FUS+MB mediated Aβ plaque clearance.

### *In vivo* 2PFM monitoring of peripheral immune cell response to FUS+MB BBB treatments

#### Animal preparation

All animal procedures were approved and conducted in compliance with the Animal Care Committee guidelines at Sunnybrook Research Institute, Canada. Male and female EGFP Wistar rats [Wistar-TgN(CAG-GFP)184ys] (310-630 g) (*n* = 15) were used in this study. Transgenic EGFP rats harbour the EGFP transgene and pCXN2 expression vector containing cytomegarovirus enhancer, chicken β-actin enhancer-promoter, and rabbit β-globin poly(A) signal 22. The GFP gene was designed to be expressed ubiquitously in this rat model, enabling monitoring of many different cell types 23.

**Figure 1:**
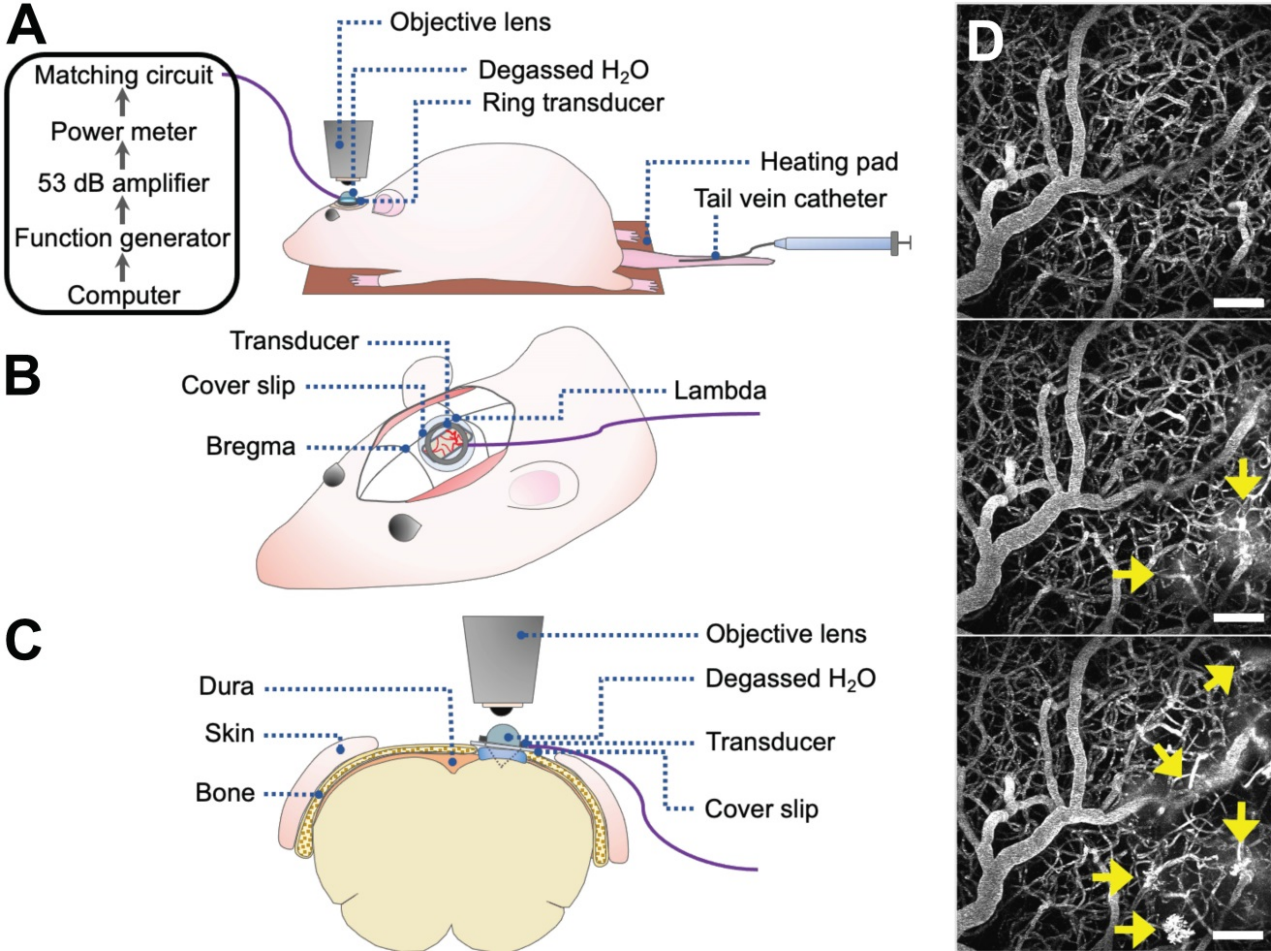
** 2PFM FUS+MB BBB experiments. (A-C)** Schematics of animal setup for monitoring FUS+MB BBB treatments using 2PFM (not to scale). **(A)** General workflow of hardware and animal setup.** (B)** Cranial window and ring transducer placement on skull. **(C)** Coronal view of experimental set up.** (D)** Maximum projection of 2PFM XYZ stack. Vasculature is visible from intravascular injection of fluorescent dextran (grey). FUS+MB induced enhanced BBB permeability is evident from the leakage of dextran into the extravascular space (arrows).

Rats underwent cranial window surgeries for acute 2PFM experiments (Figure 1). For the creation of cranial windows, rats were anesthetized with isofluorane and a core body temperature of 37°C was maintained using a heating pad with a rectal thermistor (TC-1000; CWE Inc., USA). The rat head was secured in a stereotaxic frame and a cranial window was created in the parietal bone by removing a rectangular (~ 3 × 4 mm) piece of bone using a dental drill. The dura was removed for optical clarity. Agarose gel was used to fill the removed portion of skull and dura for acoustic coupling, and a circular cover slip (5 mm diameter, no. 1 thickness; CS-5R, Warner Instruments, USA) was adhered to the cranial window using cyanoacrylate adhesive. The tail vein was cannulated with a 27 G catheter to enable intravenous injection of fluorescent dextrans and MBs.

#### Two-photon fluorescence microscopy

Imaging was performed on an FV1000MPE multiphoton laser scanning microscope (Olympus Corp., Japan) with an InSight DS tunable laser (Spectra-Physics, USA). A 25× water-immersion objective lens (XLPN25XWMP2, NA 1.05, WD 2 mm, Olympus Corp., Japan) was co-aligned with the ring transducer and water-coupled for imaging at lateral and axial resolutions of 0.994 μm/pixel and 2 μm/slice, respectively, and at an imaging speed of 2-8 μs/pixel for a maximum imaging duration of 4 h. Texas Red 70 kDa dextran (dissolved in PBS, 5 mg/kg; Invitrogen, Canada) was injected through a tail vein catheter for visualization of vasculature. EGFP cells and Texas Red dextran were excited at 900 nm. Fluorescent emissions were collected with photomultiplier tubes preceded by the following bandpass filters: 575-645 nm for Texas Red and 495-540 nm for EGFP.

After administration of Texas Red dextran, XYZT image stacks (XY: 0.994 μm/pixel, typically 512 × 512 pixels; Z: 2 μm/slice, 6-10 slices) were acquired to assess baseline immune cell dynamics. Regions-of-interest (ROIs) were centred on blood vessels that showed increased permeability following FUS+MB treatment. Imaging duration ranged from 2-4 h and was limited by increased noise from fluorescent cells reacting to the acute cranial window in the superficial layers of the cortex.

#### Focused ultrasound and microbubble treatment

An in-house manufactured lead zirconate titanate (PZT-4) cylindrical transducer (10 mm diameter, 1.5 mm thickness, 1.1 mm height) was coupled to a circular glass coverslip with cyanoacrylate adhesive, matched to a 50 Ω impedance and 0° phase load with a custom matching circuit, and driven at 1 MHz in thickness mode, producing a circular focal spot 1 mm beneath the coverslip 24. The transducer was air-backed with a droplet of degassed deionized water in the centre of the transducer to allow for use with a water-immersion objective lens (Figure 1). The transducer was driven by a computer-controlled function generator (Agilent, Palo Alto, USA), amplified with a 53 dB RF power amplifier (NP Technologies Inc., Newbury Park, USA), and transmitted through an in-house power meter and matching circuit prior to reaching the transducer. Definity^TM^ MB contrast agent (diluted 1:10 v/v in saline, 0.04 ml/kg; Lantheus Medical Imaging, MA) was administered intravenously through the tail vein catheter prior to sonication. Sonications were delivered to one location in 10 ms bursts, 1 Hz PRF, 120 s total sonication duration, with estimated *in situ* peak pressures of 0.28-0.55 MPa.

#### Vessel segmentation and permeability analysis

Two-photon fluorescence XYZT image stacks were analyzed in MATLAB (Mathworks, USA). The 575-645 nm channel (Texas Red dextran signal) in baseline images was corrected for GFP bleed-through, median filtered by 3 pixels in 3-dimensions, and contrast enhanced. Thresholding was then used to create a binarized vessel mask and vasculature was segmented.

Vessel leakage was assessed by applying the baseline mask to subsequent image stacks. Here, individual vessels with observable leakage were assessed by defining an ROI in 3D, such that the extent of leakage was captured without including adjacent vessels. For each ROI, fluorescence intensity in the intra- and extravascular regions was assessed over time and normalized to compartmental volume and baseline fluorescence, with the assumption that fluorescence intensity is proportional to concentration.

Spatiotemporal leakage was assessed by performing a Euclidean distance transform, creating a distance map from each extravascular pixel to the nearest vascular structure and removing boundary effects by truncating 20 pixels on all sides. Extravascular fluorescence was normalized to compartmental volume at each distance away from the nearest vessel, and to baseline fluorescence.

Vessel diameters were determined by taking the maximum length of the signal profile perpendicular to the local tangent. New vessels were defined whenever vessel branching occurred. Vessels were classified as arterioles, venules, or capillaries based on diameter, branching, and tortuosity patterns 25, 26.

Vascular leakage was classified as 'fast' or 'slow' based on the time it took for extravascular fluorescence intensity to plateau. 'Fast' responses rose within the first 2 min following exposure and plateaued within 10 min. 'Slow' leakage was characterized by reaching peak extravascular fluorescence after longer than 10 min, sometimes with a delay in BBB leakage onset following FUS+MB treatment.

#### Cell activity data analysis

Two-photon fluorescence microscopy image stacks were analyzed with Imaris (Bitplane, Belfast, UK). 3D reconstructions of blood vessels were created using the semi-automated Surface module. In images with high background noise, such as post-treatment images in which the intensity of dextran in the extravascular space was higher than that in the intravascular space, blood vessels were manually contoured. EGFP+ cells were detected using the semi-automated Surface module for XYZT images and Spots module for XYT images. Cell activity was automatically tracked using the Track module and manually corrected. For cell displacement measurements, a minimum of ten cells were tracked. Displacement was calculated as the distance between a tracked cells' first and last position.

To evaluate the proportion of blood vessels covered by fluorescent cells, the volume or area of blood vessels and cells were measured by creating a Surface in their respective channels.

### Identification and quantification of participating peripheral immune cells

#### Immunohistochemistry and confocal microscopy

Immediately after imaging, rats were injected with a ketamine-xylazine cocktail and transcardially perfused with ice-cold saline and freshly prepared 4% PFA in phosphate buffer (0.1 M, pH 7.2). Brains were extracted and kept in 4% PFA overnight at 4°C, transferred to 30% sucrose at 4°C until saturated, and embedded in OCT using a dry ice and ethanol slurry. Brain tissue was cut coronally into 40 μm sections using a cryostat and separated into 6 series. Brain tissue from 9 sonicated animals and 2 control animals were analysed using immunohistochemistry; 6-10 sections were analysed per animal. Sections were stored in cryoprotectant at -20°C.

Prior to staining, brain sections were washed to remove cryoprotectant (wash buffer: 0.1% Triton X-100 in PBS; 10 min × 3 washes). For antigen retrieval, sections were treated with Rodent Decloaker (Biocare Medical; diluted 1:10 in ddH2O) at 70-80°C for 7 mins, and immersed in an ice bath for 1 h 27. Sections were washed, incubated in blocking solution for 1.5 h at room temperature (blocking solution: 0.1% Triton X-100, 5% donkey serum, 1% BSA), in primary antibodies for 48 h at 4°C, washed, incubated in secondary antibodies for 2 h at room temperature, and washed. Sections were mounted using Fluoromount-G (Invitrogen, USA).

The following anti-rat primary antibodies were used: to stain blood vessels, mouse anti-rat endothelial cell antibody 1 (RECA1; MCA970R; BioRad, Irvine, USA), mouse anti-PECAM-1 (also known as CD31, ab24590), and mouse anti-glucose transporter 1 (GLUT1, ab40084); to stain astrocytes, chicken anti-GFAP (ab4674); to stain neutrophils, rabbit anti-myeloperoxidase (MPO, ab9535). The following secondary antibodies were used: goat anti-chicken Alexa Fluor 488 (ab150169, abcam; Cambridge, USA), donkey anti-rabbit Alexa Fluor 568 (ab175692), donkey anti-mouse Alexa Fluor 647 (ab150107), goat anti-chicken Alexa Fluor 647 (ab150171). Primary antibodies for MPO and RECA-1 were optimized in spleen and liver tissue sections as positive controls. All antibody company codes that begin with 'ab' were from Abcam (Cambridge, USA).

Immunofluorescence was detected using a Nikon A1 laser scanning confocal microscope (Tokyo, Japan) or Zeiss Cell Observer spinning disk confocal microscope (Oberkochen, Germany), using a 20X air or 63X oil-immersion objective lens. Z-stacks were acquired using a step-size of 2 µm or 1 µm, respectively. Images were compiled using the Bio-Formats plugin in FIJI (Fiji Is Just ImageJ 28).

#### Image processing of confocal images

Two factors were used to identify polymorphonuclear neutrophils: positive staining for MPO, and multi-lobed nuclei. This excluded eosinophils, which have multilobed nuclei but do not express MPO 29, and circulating monocytes, which may weakly express MPO prior to becoming resident tissue macrophages 30 but have ellipsoidal nuclei. Astrocytes may also express MPO but do not have multilobed nuclei 31 and are easily distinguished by their morphology.

To evaluate the number of neutrophils and relevant depth in the cortex found in FUS+MB treated and control animals, means and standard deviations were evaluated across all brain sections in each animal. Means and standard errors of the mean were calculated for controls and FUS+MB treated groups.

#### Magnetic resonance guided FUS treatment and analysis

To assess the presence of neutrophils in FUS+MB treated animals without cranial windows, 5 wild-type Wistar rats (400-450 g) underwent magnetic resonance imaging guided (MRg) FUS+MB treatment, and 5 wild-type Wistar rats (400-450 g) underwent the same procedure (anesthesia, and MB and gadolinium injections) but were not exposed to FUS. Both animals were anesthetized using isoflurane. Apart from inserting a tail vein catheter and removing the fur on the top of the head, no other surgical procedures were performed.

MRgFUS experiments were performed using the LP-100 system (FUS Instruments, Toronto, Canada), consisting of a spherically curved focused transducer, an in-house manufactured PZT fiber-optic hydrophone (Precision Acoustics Ltd, UK) positioned in a 25 mm opening in the center of the transducer, and three-axis motorized positioning system. The transducer was submerged in a tank of degassed, deionized water and matched to 50 Ω, 0° at its fundamental frequency by a matching circuit (f_0_ = 580 kHz, focal number = 0.8, diameter = 75 mm). FUS exposure was delivered at 10 ms bursts and 1 Hz burst repetition frequency, for a total duration of 120 s. The positioning system was coregistered with the animal's brain via T2-weighted images (TR = 2000, TE = 60) obtained using a 7T MRI scanner (BioSpin 7030, Bruker, Billerica, MA). Definity^TM^ MBs (diluted 1:10 v/v in saline, 0.02 ml/kg; Lantheus Medical Imaging, MA) were injected into the tail vein catheter immediately before the start of FUS exposure. One target point was chosen in the motor cortex. MRgFUS experiments were conducted using an acoustic controller to enhance BBB permeability without causing tissue damage. Specifically, acoustic pressures were increased at 8 kPa increments until the magnitude of subharmonic emissions exceeded the mean of the baseline plus 10 standard deviations of the mean, at which point pressure was automatically reduced by 50% for the remainder of the sonication duration 32. Following sonication, gadolinium-based contrast agent (0.2 ml/kg; Gadovist, Scherig AG, Berlin, Germany) was administered via tail vein catheter, and contrast enhanced T1-weighted MR images were acquired to assess BBB permeability.

Animals were perfused with ice-cold saline and fresh 4% PFA within 1 hour after FUS+MB or sham treatment. Brains were harvested, processed, cut, stained, and imaged, as described above. To compare the number of neutrophils found in the FUS+MB treated hemisphere, contralateral hemisphere, and control animals, 5 brain sections were evaluated per animal. The operator was blinded to the treatment groups.

### Role of FUS+MB recruited neutrophils in phagocytosis of Aβ

To determine whether neutrophils recruited by FUS+MB BBB treatments assisted in the clearance of Aβ plaques, 7-month-old animals of the TgCRND8 mouse model of AD were administered 3 to 5 biweekly (1 treatment every 2 weeks) MRgFUS+MB BBB treatments, targeted to the bilateral hippocampi 14. MRgFUS+MB BBB treatments were conducted as previously described. The TgCRND8 mouse model is an early onset model of AD that overexpresses a double mutant form of human APP 695 (Swedish (KM670/671NL) + Indiana (V717F) mutations) 33. By 7 months-of-age, TgCRND8 mice exhibit prolific thioflavin S-positive and dense-core plaque deposition, cognitive impairment, activated microglia and astrocytes 34, and synaptic loss 35. Four TgCRND8 animals were given biweekly FUS+MB BBB treatments, and 5 TgCRND8 animals were given sham treatment, consisting of the same preparation and anesthesia exposure but without sonication. Animals were sacrificed on the tenth week, two weeks following the fifth FUS+MB or sham treatment, at 9.5-months-old.

Animals were perfused and brain tissue was harvested as described above. For immunohistochemical analyses, the following primary antibodies were used: anti-mouse Ly6G for neutrophils (127602; BioLegend, San Diego, USA), 6F3D for Aβ plaques (Dako, Glostrup, Denmark), and chicken anti-GFAP for astrocytes (ab4674; Abcam, Cambridge, USA). Five brain sections were analyzed per animal. Stained brain sections were scanned with a confocal microscope and analyzed with FIJI.

### Statistical analyses

All statistical analyses were performed using Python.

To compare the number and depth of neutrophils in FUS+MB treated and control animals in the 2PFM experiments, the two groups were compared using a Mann-Whitney U test for unpaired non-parametric data at each depth.

In the analysis of total neutrophils counted in MRgFUS+MB BBB treated brain hemispheres ('sonicated' group, *n* = 5), contralateral hemispheres ('contralateral' group, *n* = 5), and control animals ('control', *n* = 5), group means were compared using a one-way ANOVA and Tukey's test of multiple comparisons. To compare peak BBB permeability time, blood vessel groups ('arteriole', 'venule', 'capillary') were compared using one-way ANOVAs. In TgCRND8 animals, neutrophil counts were compared in FUS+MB treated animals ('TgFUS', *n* = 4) compared to control animals ('TgCTL', *n* = 5) using a *t*-test.

Speeds of tracked intravascular cells in affected arterioles and venules were compared using a two-way ANOVA with 'time' and 'blood vessel type' as the independent groups.

Data is available upon reasonable request.

## Results

### FUS+MB BBB treatments cause an increase in BBB permeability in all blood vessel types

A total of 15 rats were monitored using 2PFM: 13 received FUS+MB treatments and 2 were no-FUS, no-MB controls. Of the 13 treated rats, 2 were excluded from analysis as the location of increased BBB permeability was outside of the FOV. An increase in BBB permeability resulting from FUS+MB exposures was successfully detected in 11/13 treated animals.

**Figure 2:**
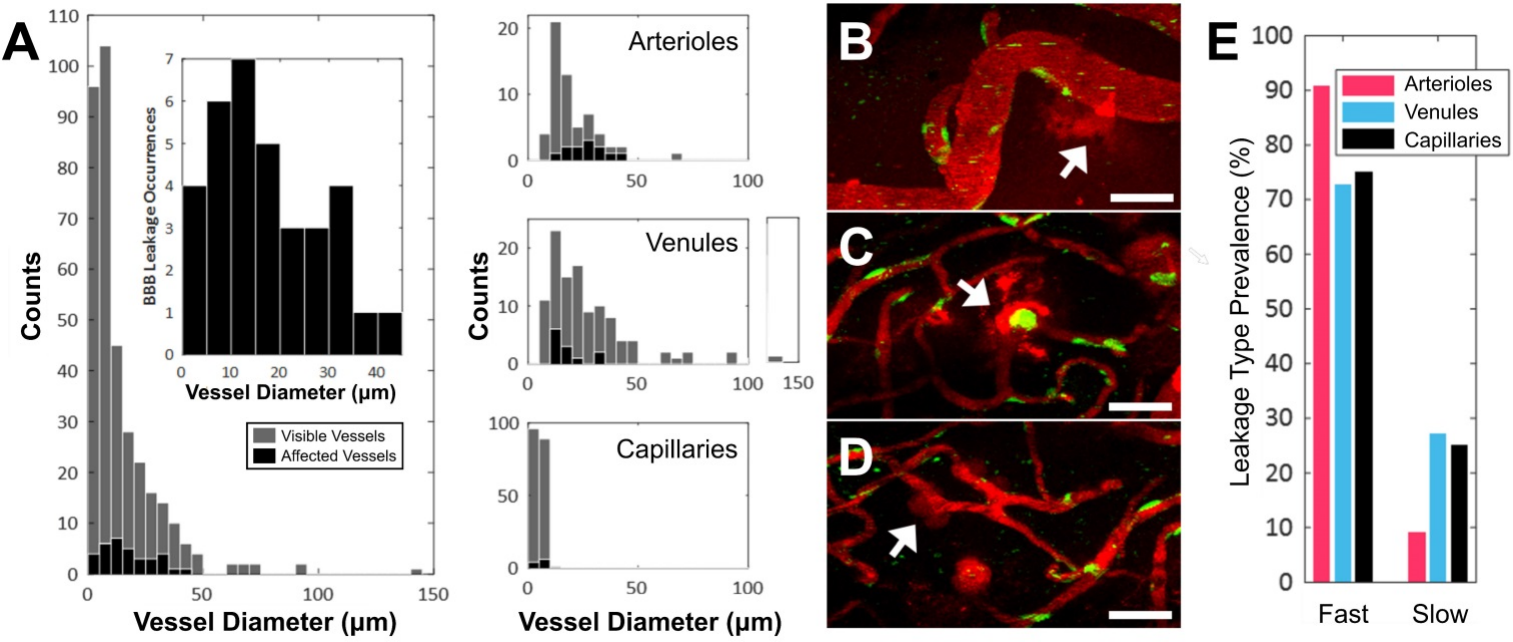
** Vascular permeability organized by blood vessel type. (A)** Histogram of enhanced BBB permeability resulting from FUS+MB exposures, by blood vessel diameter and vessel type. Representative images of FUS+MB mediated increases in BBB permeability for each blood vessel type: **(B)** arteriole, **(C)** venule,** (D)** capillary. Arrows indicate blood vessels with FUS+MB induced increases in BBB permeability. **(E)** Occurrences of 'fast' and 'slow' categorized leakage profiles according to vessel type. Blood vessel are shown in red (Texas Red dextran), and EGFP+ fluorescence cells are shown in green. Scale bars: 50 µm.

Of the 354 total blood vessel segments across the 11 treated animals that were imaged in this study, 34 blood vessels showed an increase in BBB permeability following FUS+MB treatment. Of the 34 affected blood vessels, 35% were arterioles, 35% were venules, and 30% were capillaries. The number of FUS+MB affected blood vessels represented 20% of total arteriole segments, 11% of total venule segments, and 5% of total capillary segments observed in the 11 treated animals (Figure 2A-D). FUS+MB treatments did not appear to differentially affect blood vessel types or sizes.

Fast leakage, defined as cases in which extravascular fluorescence intensity plateaued within 10 mins, was predominant in all affected blood vessel types: 91% of arterioles, 73% of venules, and 75% of capillaries (Figure 2E). Slow leakage was defined by a similar leakage onset time as fast leakage, but exhibited a slower time for extravascular fluorescence intensity to plateau (> 10 mins). In several FUS+MB affected capillaries, this was due to the presence of constrained 'clouds' of dextran that remained adjacent to FUS+MB affected capillaries for as long as 4 h post-sonication (Figure 2D, arrow). In some cases, regular spacing between dextran 'clouds' were observed (Figure 4E). This anisotropic 'dextran cloud' phenomenon was also observed in one affected venule. The retention of the shape of 'dextran clouds' over hours' of imaging suggests that the dextran may have been constrained by the extracellular matrix and/or basement membrane.

### Peripheral immune cells respond to FUS+MB BBB treatments

**Figure 3:**
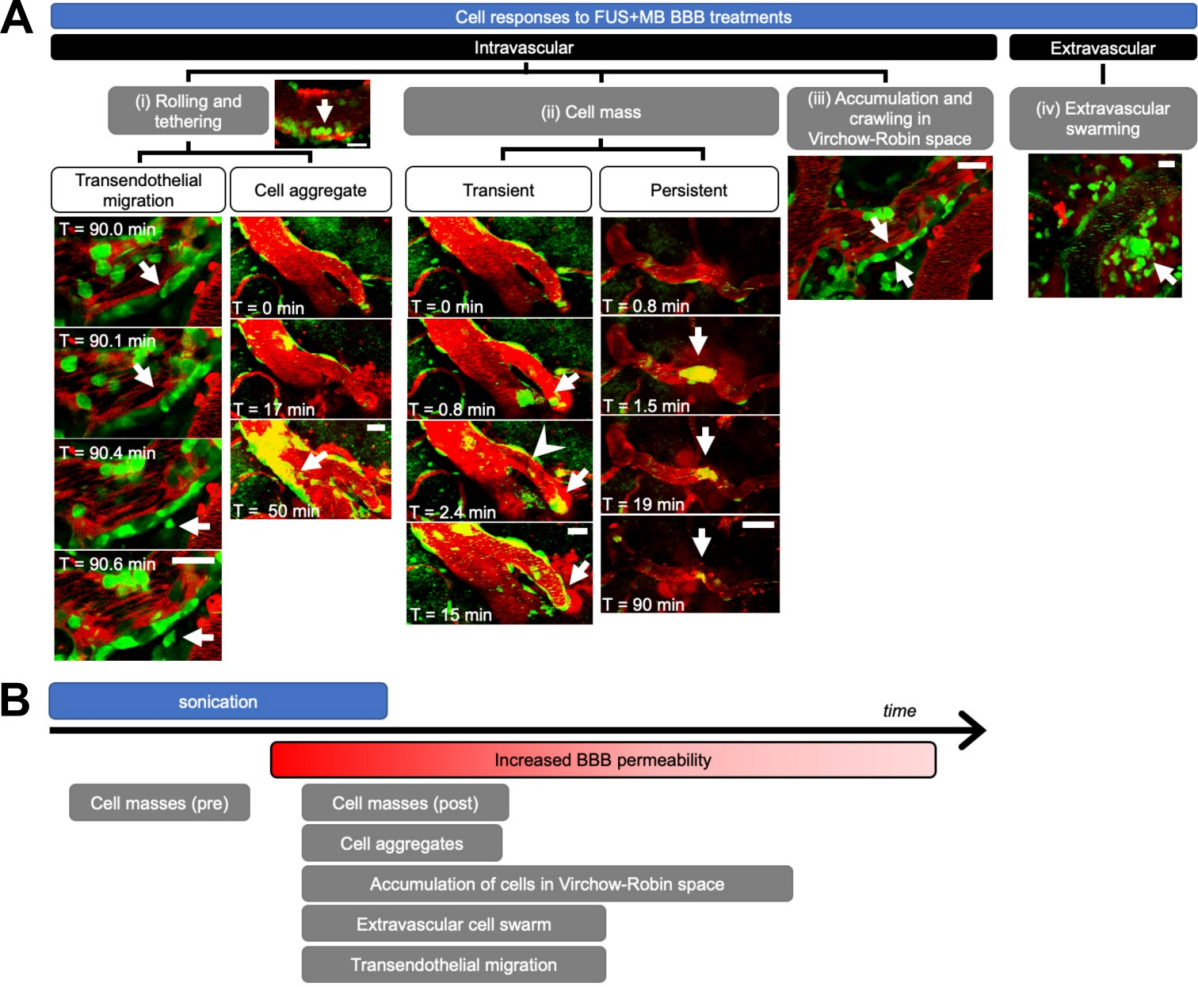
** Cell response to FUS+MB BBB treatments. (A)** Intravascular cell activity was categorized into 3 groups: **(i)** Cells (green) rolled and tethered to the luminal side of FUS+MB affected blood vessels (red), at times changing morphology and extravasating through the ECs and basement membrane to the brain parenchyma ('Transendothelial migration'). In some cases, tethered cells were observed to recruit more cells to the affected region ('Cell aggregate'). **(ii)** Groups of cells ('Cell mass', arrows) were observed to block blood vessels and affect blood flow (arrowhead), at times resolving within seconds ('Transient') or minutes ('Persistent'). **(iii)** Cells crawled and accumulated in the Virchow-Robin space of venules. **(iv)** An extravascular swarm of cells was observed in one animal following FUS+MB BBB treatment. Scale bars: 20 µm. Time is relative to the onset of FUS exposure. **(B)** Timeline of observed cell responses with respect to FUS exposure ('sonication') and onset of observed increase in BBB permeability (not to scale). FUS+MB: focused ultrasound and microbubble treatments.

Immune cell activity was observed in all blood vessel segments that exhibited increased BBB permeability after FUS+MB treatments. Intravascular cell activity was categorized into three groups (Figure 3A). Cells rolled and tethered along the luminal aspect of FUS+MB affected blood vessels, often along the same points within the vessel. Cells that adhered to the vessel wall were at times observed to extravasate to the brain parenchyma ('transendothelial migration', Supplementary Video S1) or formed 'cell aggregates' (Supplementary Video S2) by recruiting more cells to the affected area over time. Preformed groups of 'cell masses' were also observed to flow through blood vessels during sonication, and before, during, or after the onset of BBB leakage (Supplementary Video S3, S4). In contrast to the aforementioned 'cell aggregates' which increased in the number of recruited cells over the course of imaging (minutes to hours), preformed cell masses either decreased or remained constant in size, and were often observed within seconds of the onset of sonication. Preformed cell masses often appeared in blood vessel segments that showed BBB leakage, but were also observed in blood vessel segments that did not show changes in permeability. Preformed cell masses large enough to span the diameter of the lumen were observed to stop within blood vessels, stalling 'transiently' for seconds, or blocking blood flow for minutes ('persistent', Supplementary Video S5).

The accumulation and crawling of cells in the paravascular, or 'Virchow-Robin', space was observed in one venule (Supplementary Video S6). In contrast to cells in the lumen, cells in the paravascular space did not appear to be affected by blood flow, exhibiting slow crawling speeds, at times in a different direction from blood flow. In addition, the flatter morphology of these cells indicated that they were spatially constrained. Cells in the paravascular space moved too slowly to track accurately, and jitter from image acquisition and biological signals (artifacts from heartbeat, breathing) contributed more to measured cell movement than the movement of the cells themselves. Similarly, cells that were adhered to the luminal side of the vascular wall, outside of the paravascular space, did not exhibit significant changes in speed throughout 3.8 h of observation post-FUS (average speed 1.35 μm/min, data not shown). Cells observed to move at high speeds were driven by blood flow.

Extravascular cell activity resulting from FUS+MB treatment was observed in one case (Figure 3A, Supplementary Video S7). Here, motile cells accumulated and swarmed around a FUS+MB affected arteriole within 1 h following FUS exposure.

The temporal order of these cell responses with respect to FUS exposure (sonication) and BBB leakage ('onset of increase in BBB permeability') is shown in Figure 3B. Of note, cell masses were observed following FUS exposure, but even before changes in BBB permeability were observed. Although accumulation of cell aggregates, extravascular swarm, and cells crawling within the Virchow-Robin space were observed following BBB leakage, the time course of these interactions exceeded the imaging time; resolution of these events was not determined. Outstanding immune cell activity was not observed in no-FUS, no-MB, control animals (*n* = 2), who underwent identical anesthesia, cranial window surgeries, transducer placement, and imaging protocols. In these animals, fluorescent cells were observed to flow through blood vessels, and exhibited little to no endothelial adherence, confirming that the peripheral immune cell responses observed in the treated animals were due to FUS+MB BBB treatments, and not to other experimental parameters.

### FUS+MB mediated increases in BBB permeability is accompanied by the presence of cell masses, cell occlusions, and changes in blood flow

**Figure 4:**
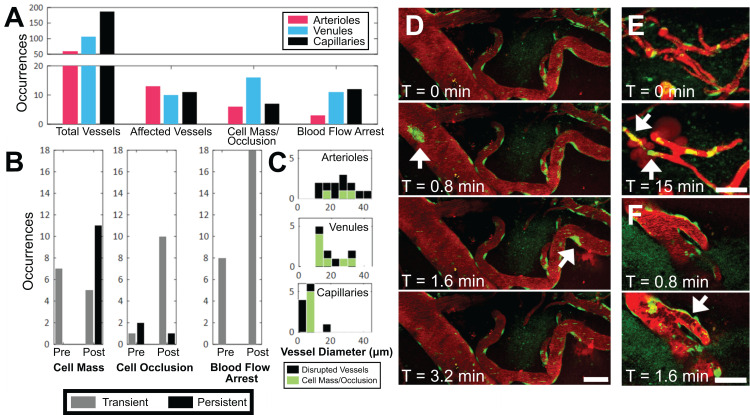
** Occurrence of intravascular cell masses, occlusions, and blood flow arrest, by blood vessel type. (A)** Number of blood vessel segments observed across all animals ('Total Vessels'), FUS+MB affected blood vessels (i.e. exhibited increases in BBB permeability, 'Affected Vessels'), blood vessels with a 'Cell Mass/Occlusion', and blood vessels exhibiting 'Blood Flow Arrest' observed, grouped by blood vessel type, and **(B)** relative to the time point at which increases in BBB permeability were observed ('Pre' or 'Post'). **(C)** Occurrences of cell masses and/or occlusions in affected blood vessels are shown in green, compared to all blood vessels in which BBB leakage was observed (black). Examples of **(D)** cell masses, **(E)** cell occlusion, and **(F)** blood flow arrest, here observed as stalled erythrocytes (dark shapes). The duration for which cell masses, cell occlusions, and blood flow arrest were observed were grouped as 'transient' (seconds) or 'persistent' (minutes), shown in **B**. Time indicated is relative to the onset of FUS exposure. Blood vessel are shown in red (Texas Red dextran), and EGFP+ fluorescence cells are shown in green. Scale bars: 50 µm.

During sonication, preformed cell masses, observed as groups of fluorescent cells that were transported as compact masses at the rate of blood flow within vasculature, were observed in all three blood vessel types (Figure 4A,D). Cell masses that were large enough to occlude the lumen of a blood vessel (Figure 4E), thereby affecting blood flow (Figure 4F), were separately identified as 'cell occlusions'. Perturbations and arrests in blood flow ('blood flow arrest') were also observed in the absence of cell occlusions, possibly due to cell occlusions that were outside of the FOV. Cell masses, cell occlusions, and blood flow arrests were observed exclusively after the onset of sonication, but were identified before ('Pre') and after ('Post') increases in BBB permeability were observed (Figure 4B).

Cell masses were observed in all three blood vessel types (Figure 4A,D). Cell masses were observed in blood vessels that did not show an increase in BBB permeability as well as ones that did. Of all blood vessels in which preformed cell masses were observed, 70% were observed after an increase in BBB permeability was observed ('Post', Figure 4B). All preformed cell masses that were observed before the onset of BBB leakage ('Pre') were 'transient' (Supplementary Video S3), lasting seconds, whereas a subset of those observed after the onset of BBB leakage ('Post') were 'persistent' for minutes to hours (Supplementary Video S4). Of note, the 'Pre' and 'Post' designations were limited by the imaging FOV: blood vessels outside of the FOV may have experienced increases in permeability, and the resulting dextran leakage into the extravascular space may not have been detected within the imaging FOV.

Cell occlusions were observed in all three blood vessel types (Figure 4A,E, Supplementary Video S5). Most cell occlusions were accompanied by blood flow arrest (Supplementary Table S1). Similar to preformed cell masses, cell occlusions were primarily observed 'Post'-BBB leakage detection (79% of cases, Figure 4B). In contrast to preformed cell masses, cell occlusions observed 'Post'-BBB leakage were primarily 'transient' (91% of cases). A cell mass or cell occlusion was observed in 23% of arterioles that showed BBB leakage, 64% of venules, and 45% of capillaries (Figure 4C). A cell mass or cell occlusion was observed in all animals that underwent a FUS+MB BBB treatment.

Blood flow arrests were identified by stationary fluorescent cells and/or non-fluorescent erythrocytes within blood vessels, and an increase in fluorescent dextran intensity in the blood vessel segment (Figure 4F). Blood flow arrests were common near points of bifurcation or in large vessels orthogonal to the propagating ultrasound wave. Perturbations in blood flow were also observed as eddies. Similar to cell occlusions, blood flow arrests were observed in all three blood vessel types (Figure 4A). Blood flow arrests were predominantly observed “Post”-BBB leakage (70% of cases, Figure 4B, Supplementary Table S1). All observed blood flow arrests were 'transient'. Only 31% of blood vessel segments in which blood flow arrest was observed showed an increase in BBB permeability, although this may be in part due to the spatial limits of the imaging FOV.

### Intravascular cell adherence to FUS+MB affected blood vessels is heterogenous

**Figure 5:**
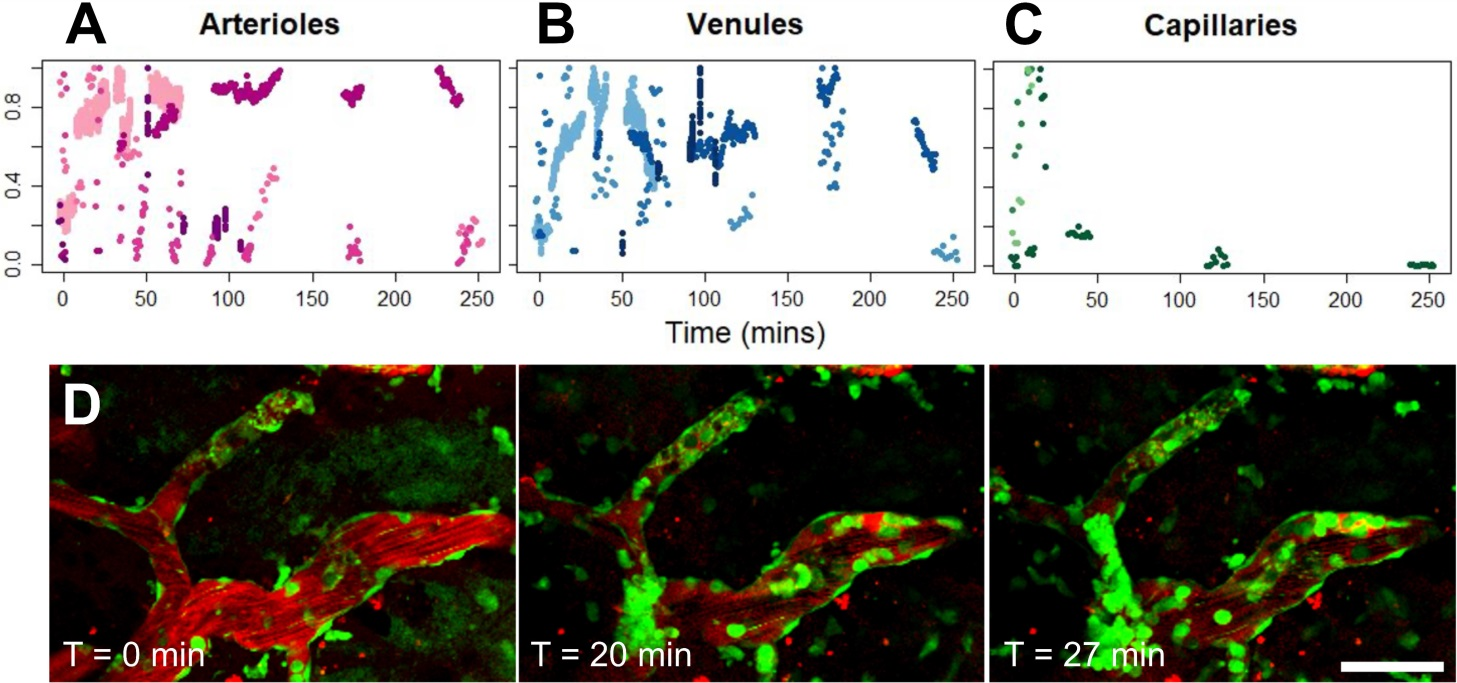
** Cell aggregate coverage of blood vessels, by blood vessel type. (A-C)** Normalized proportion of cell aggregate coverage of blood vessels in arterioles (*n* = 5), venules (*n* = 5), and capillaries (*n* = 3), where each animal is represented by a different hue. **(D)** Example of cell aggregate in a venule. Blood vessel are shown in red (Texas Red dextran), and EGFP+ fluorescence cells are shown in green. Scale bar: 50 µm.

'Cell aggregates' were identified as intravascular groups of cells that increased in size over time (Figure 3A, Supplementary Video S2). Specifically, following FUS+MB induced BBB leakage, cells were observed to tether and adhere to the luminal wall of the affected blood vessel, and recruit more cells over time. To determine whether intravascular cell aggregates displayed differential behaviours depending on blood vessel type, the proportion of blood vessel segments that were covered by cell aggregates was measured over time (Figure 5). Intravascular cell aggregate coverage of blood vessels in affected venules and arterioles showed heterogeneous responses (*n* = 5 for affected venules and arterioles, each), and did not appear to be resolved within 4 h of imaging in some cases. Intravascular cell accumulation in capillaries appeared to peak soon after sonication (*n* = 3). The speed and displacement of cells within an affected venule did not change with time. In affected capillaries, the number of intravascular cells was low, and cells were minimally motile (data not shown).

### Extravascular leukocytes swarm around an affected arteriole, increased in volume for 4 h

**Figure 6:**
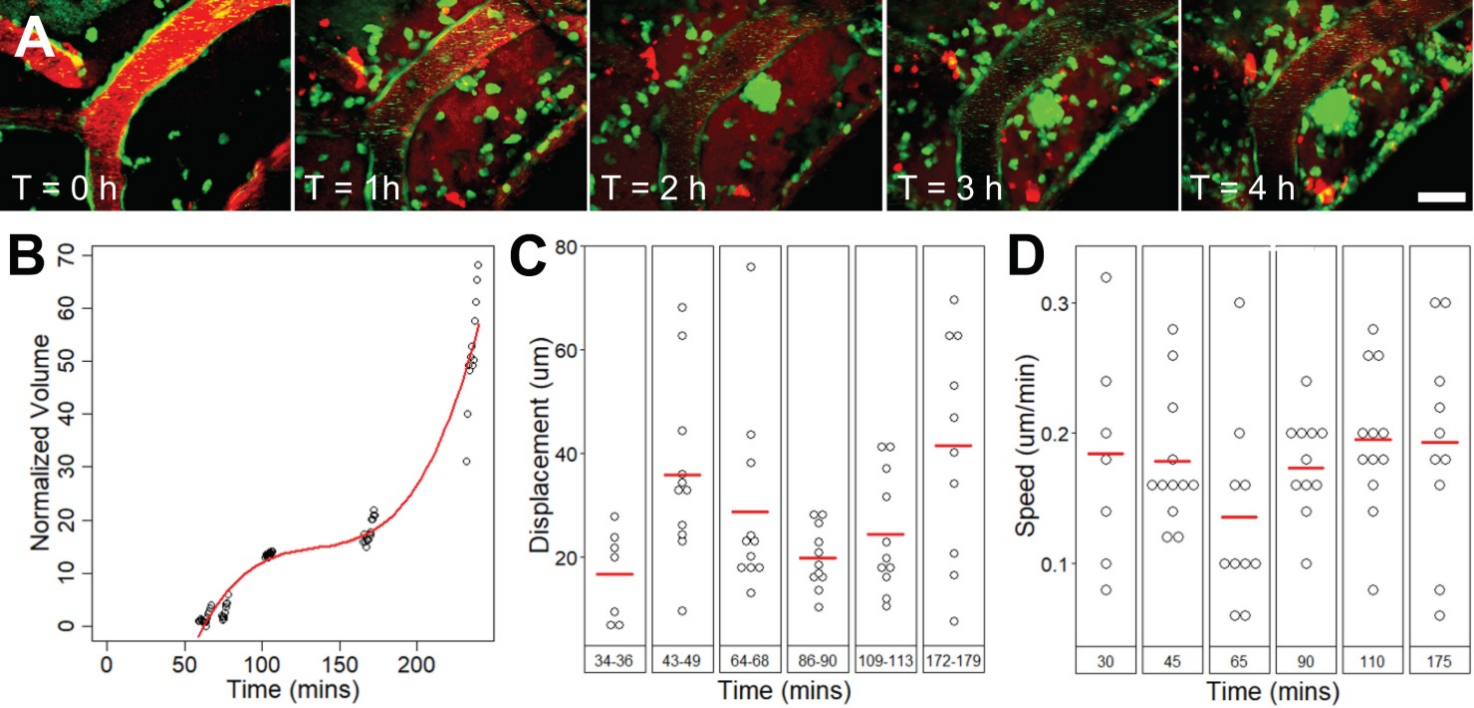
** Extravascular cell swarm.** Extravascular cell swarm around a FUS+MB affected arteriole was observed in one animal. (**A**) A mass of cells began to accumulate adjacent to an affected arteriole at T = 1 h after the onset of sonication, and increased in volume until the end of imaging. (**B**) The normalized volume, (**C**) displacement, and **(D)** speed of cells participating in the swarm, are shown. Blood vessel are shown in red (Texas Red dextran), and EGFP+ fluorescence cells are shown in green. Scale bar: 20 µm. h = hour(s).

Extravascular cell activity resulting from FUS+MB exposure was observed in one case (Figure 6, Supplementary Video S7). Here, motile cells accumulated and swarmed around a FUS+MB affected arteriole within 1 h following FUS exposure. Two types of cell activity were observed: cells that formed a dense core of cells adjacent to the arteriole, and cells that were more peripherally associated, moving rapidly into and out of the dense core of cells. In the 4 h following FUS exposure, the increase in volume of the dense core of cells approximated a cubic relationship (Figure 6B). The displacement (Figure 6C) and speed (Figure 6D) of these extravascular cells did not change appreciably over time.

### Neutrophils identified as the primary reactive cell type in the acute stages following FUS+MB mediated BBB treatments

**Figure 7:**
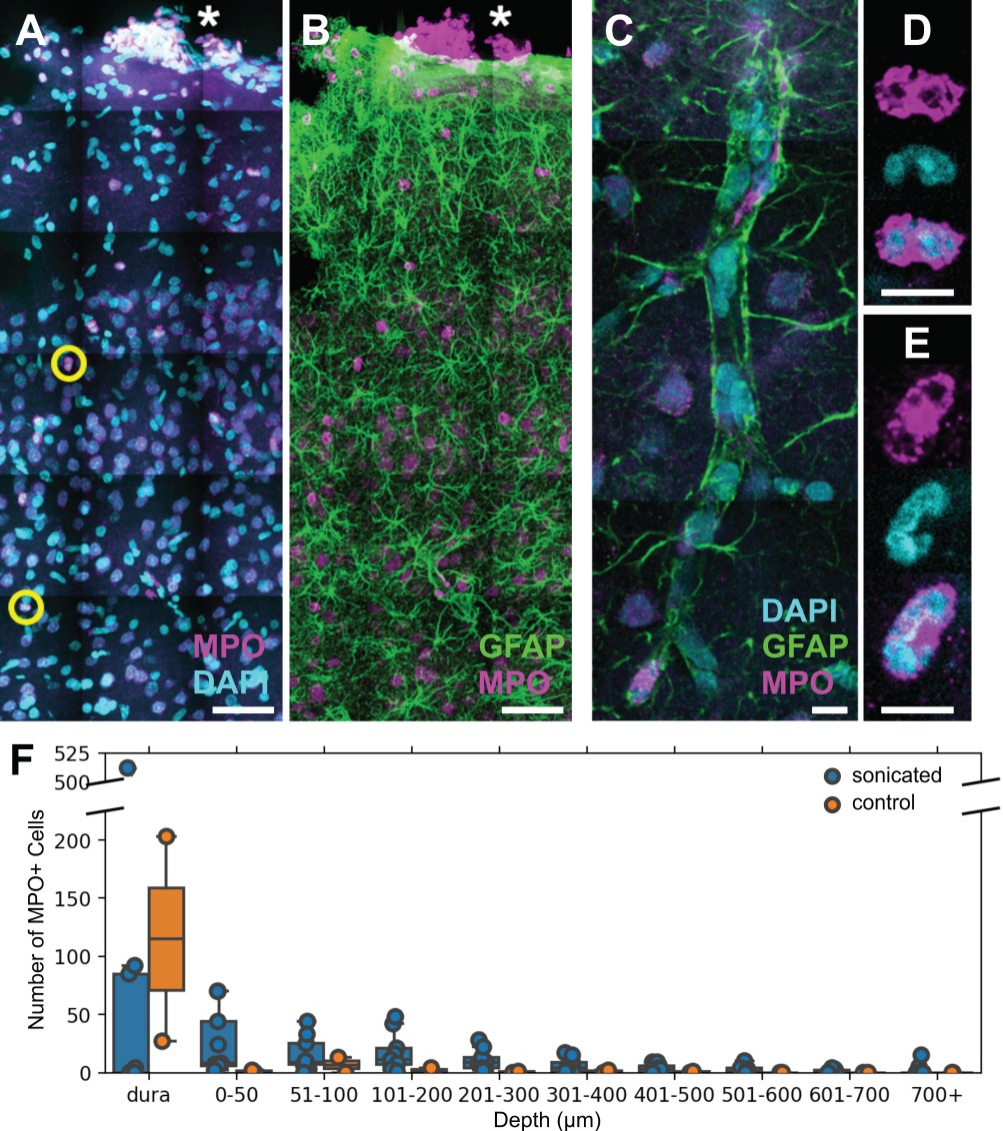
** FUS+MB BBB treatments cause neutrophil recruitment.** Polymorphonuclear neutrophils were observed in FUS+MB treated cortical tissue. **(A, B)** Brain sections were stained for neutrophils (MPO, magenta), astrocytes (GFAP, green), and nuclei (DAPI, cyan). Numerous MPO+ neutrophils were present in the dura (*), in both sonicated and control animals, likely due to the cranial window procedure. **(C-E)** Neutrophils were found within and outside of blood vessels. **(C)** A blood vessel is outlined by GFAP+ (green) astroglial processes. **(D,E)** Neutrophils were identified as MPO+ (magenta) cells containing multi-lobed nuclei (counter-stained with DAPI, cyan). **(F)** Neutrophils were more numerous and located deeper in the cortex in FUS+MB treated animals (blue), compared to control animals (orange). Scale bars: (A,B) 50 µm, (C-E) 10 µm. GFAP: glial fibrillary acidic protein, MPO: myeloperoxidase.

Based on the size, motility, and rapid response of the cells observed in the 2PFM experiments, it was hypothesized that the main population of reactive cells were neutrophils (Figure 7).

Polymorphonuclear neutrophils were identified by staining positively for MPO and exhibiting multi-lobed nuclei (Figure 7A-E). MPO is an enzymatic protein primarily produced by neutrophils, comprising ~5% of the dry mass of neutrophils 36. Brain regions affected by FUS+MBs were determined by anatomical location relative to cranial window implantation, and astrocyte reactivity 11, 13.

Neutrophils were also detected in 1 of the 2 control animals that underwent cranial window implantation but were not exposed to FUS. It was hypothesized that the presence of neutrophils could be due to the invasiveness of the cranial window surgery. Accordingly, in contrast to FUS+MB treated animals, neutrophils in the control animal were limited to the dura and superficial layers of the cortex (Figure 7F).

**Figure 8:**
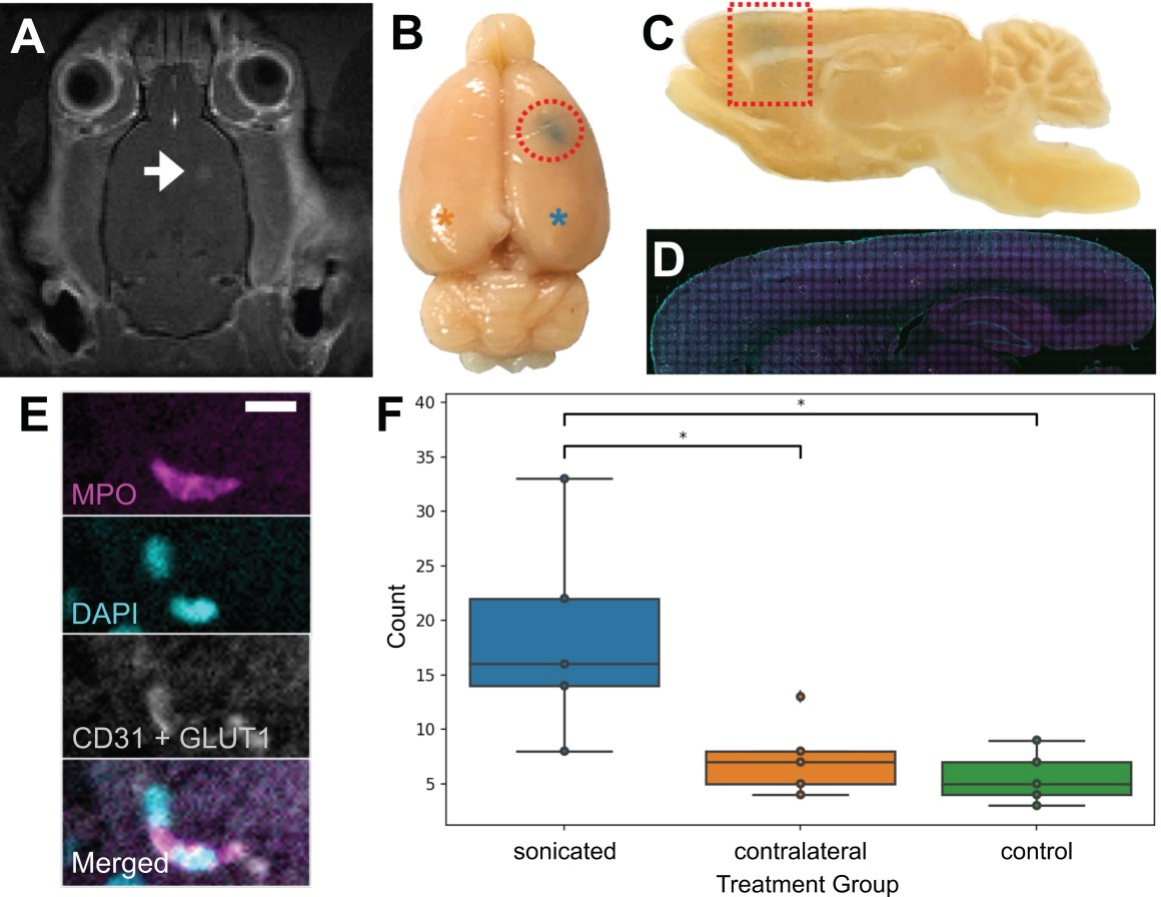
** Validation of FUS+MB induced neutrophil recruitment: MRgFUS+MB experiments.** To confirm that neutrophils were present in the brain due to FUS+MB treatments and not solely because of the cranial window surgery, MRgFUS+MB treatments, which do not require a cranial window, were administered. Areas of increased BBB permeability are evident from **(A)** gadolinium leakage in contrast-enhanced T1-weighted MRI (hyperintensity, white) and **(B)** Evans blue dye leakage in the targeted brain region (blue, emphasized by red outlines). The blue asterisk indicates the sonicated hemisphere, and the orange asterisk indicates the contralateral hemisphere (reference for F). **(C-E)** Sagittally cut brain sections were stained for neutrophils (MPO, magenta) and blood vessels (CD31 and GLUT1, white), and counterstained with DAPI (cyan). Evans blue leakage is shown by the dotted red shapes. **(F)** Sonicated hemispheres (18.6 ± 9.5, *n* = 5) harboured significantly more neutrophils compared to the contralateral hemisphere (7.4 ± 3.5, *p* = 0.03, *n* = 5), and to control animals (5.6 ± 2.4, *p* = 0.01, *n* = 5). Mean ± SD. Scale bar: 10 µm. GLUT1: glucose transporter 1, MPO: myeloperoxidase. Boxplots show median, and upper and lower quartiles.

To confirm that neutrophils were present in the brain parenchyma due to FUS+MB BBB treatments and not due to the cranial window surgeries, 5 wild-type rats underwent a single point sonication targeted to the cortex in a MRgFUS+MB BBB treatment (Figure 8), and 5 wild-type rats underwent sham treatments, consisting of the same preparation and anesthesia exposure but without sonication. In this experiment, the skull remained intact. Successful increase in BBB permeability was determined by the leakage of gadolinium in contrast-enhanced T1w MRIs (Figure 8A), and confirmed by leakage of Evans blue (Figure 8B). Animals were sacrificed within 1 hour following FUS+MB treatment. Brain tissue was separated into three groups: sonicated hemisphere, contralateral hemisphere, and hemispheres from control animals. Significantly more neutrophils were observed in the sonicated hemisphere (18.6 ± 9.5; mean ± SD) compared to control animals (5.6 ± 2.4 cells; *n* = 5 per group, *p* = 0.01), as well as to the contralateral hemisphere (7.4 ± 3.5; *n* = 5, *p* = 0.03) (Figure 8F). The majority of neutrophils observed were found within the vasculature (data not shown).

Neutrophils are capable of extruding neutrophil extracellular traps, consisting of chromatin scaffolds and enzymes including MPO 37, in response to microbial infections and in some cases of sterile inflammation 38. Immunohistochemical analyses of neutrophils did not reveal any observation of nuclear material (DAPI) outside of cell membranes, suggesting that neutrophil extracellular traps and associated neutrophil suicide were not induced by FUS+MB BBB treatments.

### Biweekly FUS+MB BBB treatments do not induce more neutrophil phagocytosis of Aβ plaques

**Figure 9:**
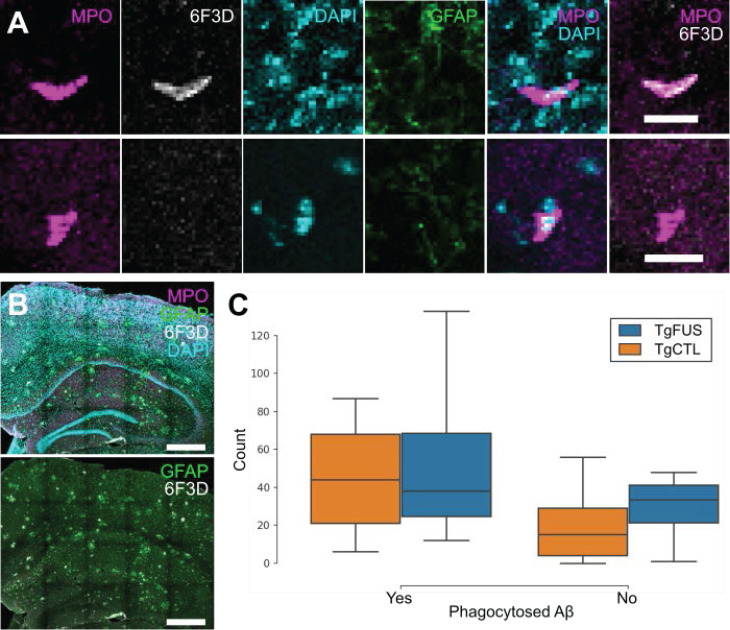
** Neutrophil counts in TgCRND8 AD mice following biweekly FUS+MB BBB treatments.** To evaluate whether neutrophils recruited to the brain due to FUS+MB BBB treatments aid in Aβ plaque clearance, animals from the TgCRND8 mouse model of AD were administered 3 to 5 biweekly treatments. Neutrophils were grouped by whether they contained phagocytosed Aβ, based on colocalization of Ly6G (neutrophils) and 6F3D (Aβ). The skull remained intact in these experiments. **(A)** Colocalization of MPO and 6F3D was used to determine whether neutrophils contained phagocytosed Aβ. Examples of neutrophils with (top) and without (bottom) phagocytosed Aβ are shown.** (B)** Neutrophils were counted within the hippocampi and cortices, which were within the focal zone of the transducer used. Sections were stained with MPO (magenta) for neutrophils, 6F3D (white) for Aβ plaques, DAPI (cyan) for nuclei, and GFAP (green) for astrocytes.** (C)** The number of neutrophils with and without phagocytosed Aβ found in FUS+MB treated animals ('TgFUS') was similar to that found in control animals ('TgCTL'). Scale bars: (A) 20 µm, (B) 500 µm. GFAP: glial fibrillary acidic protein, MPO: myeloperoxidase. Boxplots show median, and upper and lower quartiles.

To evaluate whether neutrophils recruited to the brain following FUS+MB BBB treatments aid in Aβ plaque clearance, biweekly FUS+MB BBB treatments were administered to TgCRND8 mice, and brain tissue was stained for neutrophils (anti-Ly6G) and Aβ plaques (6F3D) (Figure 9). The skull remained intact in these experiments. Neutrophils were characterized as whether they contained Aβ based on colocalization of anti-Ly6G with 6F3D staining (Figure 9A). FUS+MB treated animals did not have significantly more neutrophils with phagocytosed Aβ plaques (55.3 ± 26.9, *n* = 4) compared to control animals (45.2 ± 14.8, *n* = 5), nor did they have significantly more neutrophils without phagocytosed Aβ (TgFUS 29.0 ± 10.2, TgCTL 20.8 ± 10.1, mean ± SEM; Figure 9C).

## Discussion

**Figure 10:**
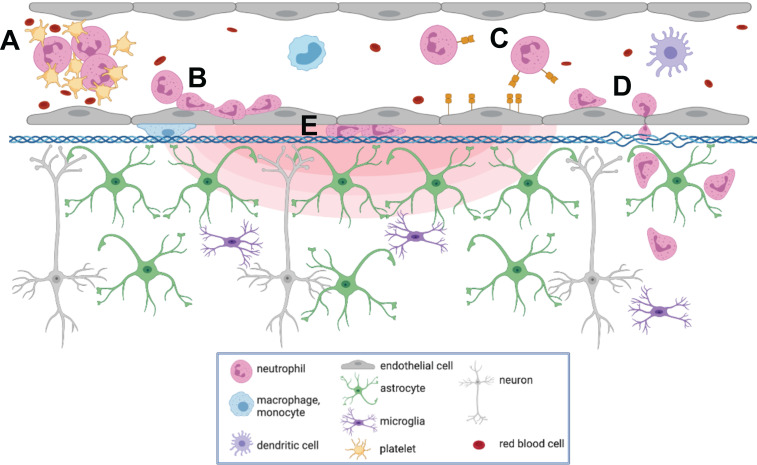
** Summary of cell responses following FUS+MB BBB treatments.** Following FUS+MB BBB treatments, an increase in vascular permeability was observed (concentric red hemi-ellipses). The following cell responses were also observed: Intravascularly, cell(s) **(A)** masses were recruited to affected blood vessels, **(B)** adhered to and aggregated on the luminal aspect of endothelial cells, **(C)** rolled, adhered to, and **(D)** extravasated across endothelial cells, and **(E)** crawled in the Virchow-Robin space of affected venules. Figure created using BioRender, not to scale.

To our knowledge, this is the first report describing immune cell responses to FUS+MB mediated BBB treatments with real-time *in vivo* imaging. The interaction of MBs with vascular endothelium during sonication recruited compact groups of cells (preformed 'cell masses') to blood vessel segments before and after the onset of increased BBB permeability, as detected by the leakage of dextran into the extravascular space. Preformed cell masses that spanned the diameter of the lumen ('cell occlusions') were observed to obstruct blood flow. Following the onset of BBB leakage, cells were observed to respond individually and as aggregates in the intravascular space. Individual cells tethered, rolled, and adhered to the luminal aspect of affected blood vessels, at times leading to the formation of cell aggregates or transendothelial migration. Cells were also observed to crawl and accumulate within the Virchow-Robin space of an affected venule, and formed an extravascular swarm around an affected arteriole. A schematic of the different cell responses observed is shown in Figure 10. One of the primary cell types participating in these responses was identified to be neutrophils. The number of neutrophils found in sonicated brain hemispheres was found to be significantly higher than that found in the contralateral hemisphere, and that in control animals, indicating that FUS+MB BBB treatments induce local recruitment of neutrophils.

To determine whether neutrophils recruited to FUS+MB affected vasculature can aid in Aβ plaque clearance, 7-month-old TgCRND8 animals were administered 3 to 5 biweekly FUS+MB BBB treatments, and sacrificed at 9.5-months-of-age, 2 weeks following the fifth treatment. This treatment regime has been previously demonstrated to result in significant reduction in Aβ plaque load 14. While neutrophils containing Aβ plaques were found, indicating an ability to phagocytose Aβ, the number of neutrophils with and without phagocytosed Aβ did not differ significantly between FUS+MB treated and control TgCRND8 animals. While this may suggest that recruited neutrophils did not aid in plaque clearance, it is also possible that the effect size of neutrophil phagocytosis of Aβ plaques may have been washed out by the time of sacrifice. The similar number of total neutrophils in the FUS+MB treated and control TgCRND8 animals suggests that the latter may be the case, as results differ from the neutrophil counts counted in animals sacrificed 1 h following FUS+MB BBB treatments (compare Figure 8F with Figure 9C). Furthermore, the immediate but transient response of neutrophils is well-described in literature: neutrophils are the first line of defense in acute inflammation, and have a short lifespan in tissue (0.75 days in mice, 5.4 days in humans 39). In addition, extravasated neutrophils may return to circulation through reverse transendothelial migration 40.

Despite their limited effect on Aβ clearance, FUS+MB induced recruitment of neutrophils may aid in Aβ clearance through downstream recruitment of other leukocytes. Specifically, neutrophils can recruit blood monocytes to inflammatory sites through secretion of soluble factors such as C-C chemokines 41. Recruited monocytes and macrophages have longer lifespans in tissue 42 and can emigrate to the lymphatics system 43. As such, monocytes and macrophages may have a greater capacity than neutrophils in clearing Aβ 44. For example, depletion of monocytes resulted in worsened outcomes following parasite infection, whereas depletion of neutrophils did not result in any pathological changes, suggesting that monocytes, but not neutrophils, may play a protective role 45.

The cell responses observed in this study, such as transendothelial migration of leukocytes and leukocyte aggregate adhesion, are known components of acute inflammation 46. Transcriptional changes indicative of an acute inflammatory response following FUS+MB BBB treatments have been previously reported, including an upregulation of genes that support immune cell migration (*Sele*, *Cxcl1*, *Ccl7*) 19, 20, 47. This study provides evidence that the acute inflammatory response, observed as leukocyte recruitment, begins during FUS exposure, whereas the earliest time point measured for previous studies were 5 mins 48, and 6 h following FUS+MB treatments 19. The speed of cell responses led to the hypothesis that neutrophils are a key participating leukocyte, as they are the most abundant leukocytes in circulation, as well as the first immune cells to transmigrate to areas of inflammation through endothelial selectin-dependent tethering and rolling 49. However, the heterogeneity of cell responses observed suggests that FUS+MB BBB treatments may induce a multistep inflammatory response, involving several types of leukocytes that lead to increases in vascular permeability and recruitment 46, 50. Other phagocytic leukocytes with longer lifespans in tissue than neutrophils may also be aiding in FUS+MB induced Aβ clearance. Different cell types may also respond differently according to the extent of enhanced BBB permeability achieved.

The cell aggregates observed in this study may be comprised of platelets and leukocytes. In addition to myeloid leukocytes, platelets are also capable of rolling, adhering, and aggregating on activated endothelium 51. As part of the innate immune response, platelets can form aggregates with neutrophils and circulating monocytes, and promote neutrophil tethering to activate ECs through expression of selectins and inflammatory cytokines and chemokines 52. Platelets can also interact with dendritic cells, which can lead to recruitment of T cells 50. Recruitment of leukocytes and leukocyte-platelet aggregates may also contribute to increased microvascular permeability 46.

In one case, an extravascular swarm of leukocytes was observed following BBB leakage. The activity and morphology of these cells resembled the rapid chemotaxis of neutrophils to sites of sterile inflammation 53, suggesting that FUS+MB treatment in this case may have caused vascular damage. Similarly, macrophage infiltration into the brain have been observed following FUS+MB treatments using parameters designed to cause tissue damage, but not if sonication parameters and MB dosages for reversible BBB treatments that do not produce overt tissue damage were used 18, 20, 54.

Alterations in blood flow were observed during and after FUS+MB BBB treatments, at times accompanied by cell occlusions. Reversible local disturbances in blood flow during sonication are likely due to radiation force of the propagating ultrasound wave. Similar phenomena, described as 'blood cell stasis' or 'parathrombosis', have been previously observed in human blood *in vitro* 55, and chick embryos *in vivo* 56, 57. Persistent stalls in capillaries may affect short-term memory function 58, and should be further examined in the context of FUS+MB treatments.

An anisotropic distribution of dextran diffusion was observed surrounding FUS+MB affected blood vessel segments. Increases in fractional anisotropy, a measure of diffusion anisotropy, have been observed in grey matter areas with immunoglobulin leakage in a rodent model of acute reversible encephalopathy 59. It is possible that the anisotropy observed on the microscopic scale in these FUS+MB BBB 2PFM experiments may be detectable in fractional anisotropy measurements taken from diffusion tensor imaging MRI scans, as immunoglobulin extravasation has been observed following FUS+MB BBB experiments as well 11. Such measurements would enable more global analyses of leakage kinetics following FUS+MB BBB treatments. The observed anisotropic distribution and constraint of dextran around blood vessels may be due to heterogeneities in the vascular wall and suggests the involvement of the extracellular matrix and/or basement membrane. For example, chronic hypertension, which leads to an increase in extracellular matrix proteins, including collagen 60, is the main vascular risk factor for small vessel disease, which is associated with reduced fractional anisotropy 61-63. Furthermore, remodelling of the extracellular matrix can be achieved by immune cells: neutrophils are a source of matrix metalloproteinases 64, which can degrade the extracellular matrix, and have been correlated with reduced type IV collagen expression following stroke 65. Analysis of extracellular matrix proteins following FUS+MB BBB treatments may reveal insight into leakage kinetics and the extent of immune cell infiltration.

This study may also offer insight into purported clearance mechanisms observed after FUS+MB BBB treatments. The intravascular accumulation of cells within the Virchow-Robin space of an affected venule may also support the hypothesis that FUS+MB BBB treatments affect the glymphatic clearance pathway 66. In addition, the marginal increase in the number of neutrophils in the contralateral hemisphere compared to control animals suggests that FUS+MB BBB treatments result in neutrophil recruitment in a wider area than observed changes in vascular permeability, or that recruited neutrophils have a high capacity for migration. Further investigations into the cell types recruited by FUS+MB BBB treatments and the spatial and temporal extents of treatment effects are recommended.

This study provided evidence that neutrophils respond to FUS+MB BBB treatments immediately after the onset of FUS exposure. Immune responses to FUS+MB BBB treatments were observed sooner than previously reported, and may be triggered from interactions of MB oscillations with vascular walls before increases in BBB permeability were observed. While the short lifespan of neutrophils in tissue and evidence of their return to circulation suggests that their effects following FUS+MB BBB treatments may be transient, their ability to recruit other leukocytes with longer lifespans in tissue, such as monocytes, may lead to the downstream Aβ clearance observed in previous FUS+MB AD studies. This study adds to the body of literature that points to the necessity of controlling FUS+MB treatments through optimal parameter selection and real-time monitoring, and reveals aspects of the peripheral immune system that should be explored when considering future studies involving FUS+MB BBB treatments in neuroinflammatory diseases or in conjunction with drug delivery.

## Supplementary Material

Supplementary video/movie S1-S7 and table S1.

## Abbreviations

2PFM: two-photon fluorescence microscopy
Aβ: β-amyloid
AD: Alzheimer's disease
FUS: focused ultrasound
GLUT1: glucose transporter 1
MB: microbubbles
MPO: myeloperoxidase
MRgFUS+MB: magnetic resonance guided focused ultrasound and microbubble
MRI: magnetic resonance imaging
ROI: region(s)-of-interest
