# Supplementary material for: Neutrophil recruitment and leukocyte response following focused ultrasound and microbubble mediated blood-brain barrier treatments
Source: Theranostics. 2021 Jan 1;11(4):1655–71. doi: 10.7150/thno.52710 (PMC7778596; doi:10.7150/thno.52710)
Supplement: Supplementary file 1 — Supplementary video/movie S1-S7 and table S1. [file thnov11p1655s1.zip › supplementary2_thno.pdf]

Supplementary Materials

Cell masses, occlusions, and blood flow arrests: Individual subjects								
			Cell Mass		Occlusion		Blood Flow Arrests	
Animal ID	Vessel Size (um)	Class	PRE	POST	PRE	POST	PRE	POST
609	32.02	A		persist				
621	27.42	A	transient					
621	16.64	A		persist		transient		transient
621	43.58	A	transient					transient
621	30.5	A	transient					transient
623	31.65	A		transient				
527	5.97	C		persist	transient		transient	
527	6.45	C				persist		transient
621	8.92	C		transient		transient		transient
621	4.85	C						transient
621	5.66	C				transient		transient
526	4.45	C					transient	
527	8.45	C	transient					
527	4.77	C	transient					
621	4.71	C				transient		transient
621	6.83	C						transient
621	10.86	C						transient
621	4.97	C						transient
621	5.8	C						transient
621	4.6	C						transient
330	14.86	V			persist		transient	
426	15.44	V		persist				
520	12.95	V		transient		transient		transient
526	12.1	V				transient		transient
609	28.72	V		persist				
621	12.31	V		transient		transient		
330	41.67	V		persist				
330	38.75	V		persist				
426	38.38	V		persist		transient	transient	
426	23.57	V						transient
426	21.9	V						transient
426	14.24	V		persist		transient	transient	
426	21.97	V	transient				transient	
426	13.52	V			persist		transient	
526	63.07	V		transient			transient	
526	19.5	V		persist		transient		transient
621	74.06	V	transient					
623	30.24	V		persist				

Supplementary Table S1. Cell mass, cell occlusion, and blood flow arrest data from all animals.
'Pre' indicates that an event was observed before increases in BBB permeability were detected, 'Post' indicates that an event was observed after increases in BBB permeability were detected.

Supplementary Video S1. Transendothelial migration observed following FUS+MB BBB treatment.
A cell is observed extravasating from the intravascular to the extravascular space, following FUS+MB BBB treatment. Frames are shown in hh:mm following the onset of FUS exposure.

Supplementary Video S2. Cell aggregates observed following FUS+MB BBB treatment.
Cells were observed to aggregate on the luminal surface of blood vessels following FUS+MB BBB treatments. Such 'cell aggregates' were observed to increase in size over time. Frames are shown in hh:mm following the onset of FUS exposure. Frames are shown in hh:mm following the onset of FUS exposure.

Supplementary Video S3. Cell masses (transient) observed following FUS+MB BBB treatment.
Preformed cell masses, defined as groups of fluorescent cells that were transported as compact masses at the rate of blood flow within vasculature, were observed following FUS+MB BBB treatments. In contrast to 'cell aggregates', which increase size over time, cell masses were already in 'preformed' groups at the time of observation. Here, transient cell masses (arrows) are observed before increases in BBB permeability were observed (arrowheads). Frames are shown as 'pre-FUS', indicating before FUS exposure, or in mm:ss following the onset of FUS exposure.

Supplementary Video S4. Cell masses (persistent) observed following FUS+MB BBB treatment.
Preformed cell masses, defined as groups of fluorescent cells that were transported as compact masses at the rate of blood flow within vasculature, were observed following FUS+MB BBB treatments. In contrast to 'cell aggregates', which increase size over time, cell masses were already in 'preformed' groups at the time of observation. Here, a persistent cell mass is observed in an affected blood vessel within seconds of BBB leakage. Frames are shown in mm:ss following the onset of FUS exposure.

Supplementary Video S5. Cell occlusion and blood flow arrest observed following FUS+MB BBB treatment.
Cell occlusions were observed as cell masses that spanned the diameter of blood vessels, thereby affecting blood flow. Following FUS+MB BBB treatment, BBB leakage is observed (arrowheads), and cell masses can be observed in affected blood vessels. A cell occlusion resulting in blood flow arrest is emphasized by the arrow. Frames are shown in mm:ss following the onset of FUS exposure.

Supplementary Video S6. Cells crawling in the Virchow-Robin space observed following FUS+MB BBB treatment.
In one animal, cells were observed to crawl within the Virchow-Robin space following FUS+MB BBB treatment. This was evident from the difference in the speed and direction of cell movement relative to blood flow, as well as from the flatter morphology of these cells, suggesting spatial constraint. Frames are shown in mm:ss following the onset of FUS exposure.

Supplementary Video S7. Extravascular swarming observed following FUS+MB BBB treatment.
In one animal, extravascular cell swarming was observed around an affected arteriole following FUS+MB BBB treatment. Two types of cell activity were observed: cells that formed a dense core of cells adjacent to the arteriole, and cells that were more peripherally associated, moving rapidly into and out of the dense core of cells. The activity of these cells resembled rapid chemotaxis of neutrophils to sites of sterile

inflammation, suggesting that FUS+MB treatment may have resulted in vascular damage in this case. Frames are shown in hh:mm following the onset of FUS exposure.
